# Simultaneous purification of DNA and RNA from microbiota in a single colonic mucosal biopsy

**DOI:** 10.1186/s13104-016-2110-7

**Published:** 2016-06-28

**Authors:** Aina E. F. Moen, Tone M. Tannæs, Simen Vatn, Petr Ricanek, Morten Harald Vatn, Jørgen Jahnsen, IBD-Character Consortium

**Affiliations:** Division of Medicine, Department of Clinical Molecular Biology (EpiGen), Akershus University Hospital , Lørenskog, Norway; Institute of Clinical Medicine, University of Oslo, Oslo, Norway; Division of Medicine, Department of Gastroenterology, Akershus University Hospital , Lørenskog, Norway

**Keywords:** Microbiota, RNA, DNA, IBD

## Abstract

**Background:**

Nucleic acid purification methods are of importance when performing microbiota studies and especially when analysing the intestinal microbiota as we here find a wide range of different microbes. Various considerations must be taken to lyse the microbial cell wall of each microbe. In the present article, we compare several tissue lysis steps and commercial purification kits, to achieve a joint RNA and DNA purification protocol for the purpose of investigating the microbiota and the microbiota-host interactions in a single colonic mucosal tissue sample.

**Results:**

A further optimised tissue homogenisation and lysis protocol comprising mechanical bead beating, lysis buffer replacement and enzymatic treatment, in combination with the AllPrep DNA/RNA Mini Kit (Qiagen, Hilden, Germany) resulted in efficient and simultaneous purification of microbial and human RNA and DNA from a single mucosal colonic tissue sample.

**Conclusions:**

The present work provides a unique possibility to study RNA and DNA from the same mucosal biopsy sample, making a direct comparison between metabolically active microbes and total microbial DNA. The protocol also offers an opportunity to investigate other members of a microbiota such as viruses, fungi and micro-eukaryotes, and moreover the possibility to extract data on microbiota and host interactions from one single mucosal biopsy.

**Electronic supplementary material:**

The online version of this article (doi:10.1186/s13104-016-2110-7) contains supplementary material, which is available to authorized users.

## Background

Crohn’s disease (CD) and ulcerative colitis, collectively known as inflammatory bowel diseases (IBD), are characterized by chronic intestinal inflammation with huge impact on quality of life. The actual cause of IBD remains unsolved. It is likely, however, that the chronically recurring episodes of inflammation in the human bowel are related to a complex interaction between various environmental factors, a hereditary predisposition for these diseases and the gut microbiota [1, 2]. Host genetics play a key role, but genetic defects cannot explain the increasing prevalence of IBD in recent years. One theory gaining support is that IBD results from, or is maintained by, a dysbiosis of the gut microbiota [3]. The human intestinal tract harbors a diverse and complex microbial community playing a central role in human health [4]. The disruption of the delicate balance between the microbial community and its host is thought to contribute to IBD pathogenesis. Unrevealing the inhabitants of the gut microbiota has been a goal for large research groups as the European MetaHIT (http://www.metahit.eu/) [5] and the US Human Microbiome Project (http://commonfund.nih.gov/hmp/) [6]. Both projects have primarily focused on investigating the microbial diversity of fecal samples, as is also the focus of many other research groups [7]. In IBD, investigating fecal samples from affected persons is important and valuable, but as the disease includes the affection of colonic tissue, the mucosal microbiota is of high importance. Microbial communities that reside on the surface of the intestinal mucosa encounter an environment distinct from the luminal environment, and a recent study from Tang and coworkers [8] show that the mucosal microbiota may be a reservoir for species that contributes to disease activity in colitis. A recent study on new-onset CD also confirms the superiority of using mucosal biopsies as opposed to stool when searching for potential biomarkers, as presence or absence of bacterial taxa, for disease diagnosis [3].

Nucleic acid purification methods are of importance when performing microbiota studies, and especially when analysing the intestinal microbiota, as we here find a wide range of different microbes. Various considerations must be taken with regard to lysing the microbial cell wall of each microbe. The lysis step during nucleic acid purification is thus an important step that could represent a large source of bias depending on the protocol used [9]. Studies have shown that the choice of DNA purification methods, and more importantly the choice of pre-homogenization procedures, has a large impact on the resulting microbiota composition and diversity [10, 11]. When assessing the success rate of a purification method, the method’s ability to purify ‘hard-to-lyse’ microbes, such as the Gram-positive Firmicutes, is often used for evaluation [12, 13]. Microbiota studies analysing both microbial RNA and DNA purified from mucosal biopsies have been performed, but in these studies RNA and DNA is purified from different mucosal biopsies [14, 15]. The aim of the present study is to purify both RNA and DNA for the purpose of investigating the metabolically active members of the microbiota, the total members of the microbiota and the microbiota-host interactions in a single colonic mucosal sample. To achieve the aim we have optimised a joint RNA and DNA purification protocol of a commercial nucleic acid purification kit. The resulting optimised protocol has been assessed and evaluated against standard RNA and DNA purification methods.

## Methods

### Nucleic acid purification

Colonic mucosal biopsies, 2–3 mm in diameter, were collected from two patients undergoing planned colectomy at Akershus University Hospital, and from three patients undergoing colonoscopy as part of the EU-project IBD-Character (EU ref no 305676). Prior to the colectomy and colonoscopy the patients performed a bowel cleansing by Picoprep (Ferring Legemidler AS, Oslo, Norway) according to manufacturer’s instructions. All biopsies were immediately placed in Allprotect Tissue Reagent (Qiagen, Hilden, Germany) and stored according to manufacturer’s instructions. For each of the different protocols tested, one to two biopsies from each patient were used due to a limited number of biopsies (Table 1).

**Table 1:** Purified DNA and RNA quantity and quality data and subsequent analysis

Comparison^a^	Patient	Protocol	Quantity DNA (ng/µl)	Quality DNA (260/280)	Quantity RNA (ng/µl)	Quality RNA (RIN)	cDNA synthesis^b^	Cloning and sequencing	
								cDNA	DNA^c^
1	Patient 1	Protocol 1	540	1.89	48.34	N/A			X
	Patient 1	Protocol 2	429	1.89	97.67	8.3	X (T)		
	Patient 1	DNA kit1	258	1.90					X
	Patient 1	DNA kit2	353	1.88					X
	Patient 1	DNA kit3	219	1.88					X
	Patient 2	Protocol 1	366	1.88	38.95	N/A			
	Patient 2	Protocol 2	200	1.92	85.12	8.5	X (T)		
	Patient 2	DNA kit1	117	1.91					
	Patient 2	DNA kit2	389	1.87					
	Patient 2	DNA kit3	551	1.86					
2	Patient 3	Qiazol, phase separation, kit column			140.53	8.5			
	Patient 3	Protocol 2			109.82	8.7	X (T)		
	Patient 3	Qiazol, phase separation, kit column			95.21	8.5			
	Patient 3	Protocol 2			102.2	8.9	X (T)	X	
3	Patient 4	Protocol 2	375	1.89	116.96	8.1			X (T)
	Patient 5	Protocol 2	521	1.88	56.26	7.3			X (T)
	Patient 4	Protocol 3	274	1.92	170.42	7			X (T)
	Patient 5	Protocol 3	353	1.90	102.27	7.3			X (T)

^a^Three separate comparisons of purification protocols were performed due to limited number of biopsies

^b^X = Sample being subjected to cDNA synthesis, T = Technical replicates

^c^X = Sample being subjected to cloning and sequencing, T = Technical replicates

**Fig. 1:**
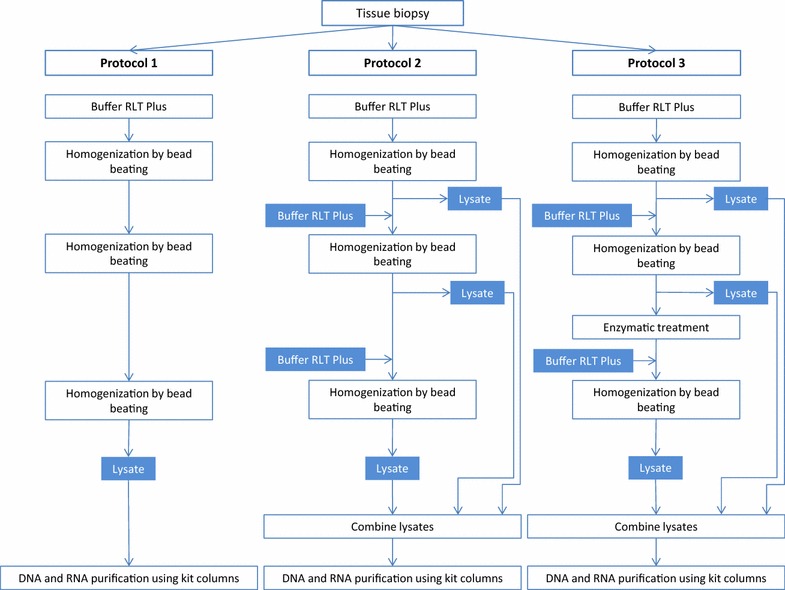
Protocol flowchart. Flowchart of the experimental set-up of protocol 1, 2 and 3

Total RNA and DNA were purified from colonic mucosal tissue using the AllPrep DNA/RNA Mini Kit (Qiagen). Manufacturer’s instructions were followed with the exception of the lysis steps, where three tissue lysis protocols (Protocol 1, 2 and 3) were performed and evaluated (Fig. 1; Additional file 1). For RNA purification, DNase treatment was included and performed on column as described in the AllPrep DNA/RNA Mini Kit protocol. RNA and DNA were eluted using 40 µl nuclease free water (NFW) and stored at −80 and −20 °C, respectively.

The resulting RNA quantity and quality data from the tested protocols were compared to RNA quantity and quality data obtained using a standard RNA purification protocol combining Qiazol, phase separation and kit column based purification [16] (Table 1; Additional file 1). The resulting DNA quantity and quality data were compared to DNA quantity and quality data from a combination of mechanical and enzymatic pre-treatments as recommended by Qiagen for lysis of Gram-positive bacteria, followed by purificaton with AllPrep DNA/RNA Mini Kit (kit 1), the QIAamp DNA Stool Mini Kit (kit 2) and the DNeasy Blood & Tissue Kit (kit 3) (Table 1; Additional file 1). For all three purification kits the manufactures instructions were followed after pre-treatment. DNA was eluted using 40 µl NFW and stored at −20 °C.

**Fig. 2:**
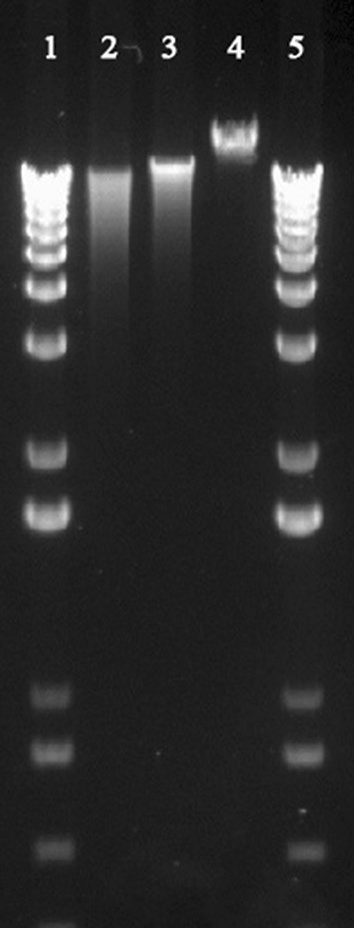
DNA quality. Gel electrophoresis of genomic DNA (1 % agarose gel in 1xTBE, 70 V, running time 2.5 h) *1* and *5* 1 kB Plus DNA ladder (Invitrogen, Thermo Fisher Scientific, Waltham, MA, USA); *2* Protocol 1 (100 ng); *3* Protocol 2 (100 ng); *4* Lambda DNA-48 kB (New England Biolabs, Ipswich, MA, USA)

The concentration of the RNA and DNA samples were assessed using NanoDrop ND-1000 spectrophotometer (Thermo Fisher Scientific, Waltham, MA, USA). For RNA quality the RNA integrity number (RIN) was tested using the Agilent 2100 Bioanalyzer (Agilent Technologies Inc., Santa Clara, CA, USA) and the 2100 Expert Software. The assay and reagent kit used were Eukaryote Total RNA Nano Series II and the Agilent RNA 6000 Nano Kit. For DNA the quality was obtained using the ratio 260/280 for assessing the purity of the samples. The molecular size of the genomic DNA was measured in a 1 % agarose gel in 1xTBE, 70 V, running time 2.5 h (Fig. 2).

### cDNA synthesis

cDNA was synthesised from 200 ng RNA using AccuScript High Fidelity 1st Strand cDNA Synthesis Kit (Agilent Technologies Inc.) according to the manufacturer’s instructions. Both gene specific primer (926R [17]) and random hexamers were tested, with technical replicates, on RNA purified using protocol 2 and tissue biopsies from three patients. Two RNA samples were run in the absence of reverse transcriptase to assess the degree of contaminating genomic DNA. To verify synthesis of microbial and human cDNA and to assess possible contaminating genomic DNA real-time PCR was performed using ABI Prism 7900HT Real Time PCR System (Applied Biosystems, Thermo Fisher Scientific, Waltham, MA, USA) and the software system SDS 2.4 (Applied Biosystems). The primers used for microbial 16S cDNA amplification were 357F and 926R [17], and for human cDNA amplification ACTB-F: 5′-GGT GTT TGT CTC TCT GAC TAG-3′, and ACTB-R: 5′-TGT CAC ACG AGC CAG TAT TAG-3′ [18]. The PCR amplifications were performed in triplicates using a 20 µl final reaction mixture containing 10 µl Power SYBR Green PCR Master mix (Applied Biosystems), 4 µl primer mix (5 µM), 4 µl NFW and 2 µl template. Default cycling conditions were used (Applied Biosystems)(Additional file 2).

### Cloning and sequencing of 16S rRNA and 16S cDNA

The 16S rRNA genes and 16S cDNA were amplified using primers 357F and 926R [17], HotMaster Taq DNA polymerase (5 PRIME GmbH, Hilden, Germany) and the following cycling conditions: Initial denaturation 94 °C (2 min), 94 °C (30 s) 58 °C (30 s) 65 °C (45 s), 30 cycl., 65 °C (7 min). Cloning of the amplified products were performed using TOPO^®^ TA cloning (Life Technologies, Thermo Fisher Scientific, Waltham, MA, USA) according to manufacturer’s instructions. Positive clones were subjected to PCR amplification using M13 primers from the cloning kit and cycling conditions according to manufacturer’s instructions. The PCR products were sequenced using Big Dye v/1.1, primers 357F and 926R [17] and ABI3130xl (Applied Biosystems) according to manufacturer’s instructions.

The 16S rRNA gene cloning and sequencing was performed using DNA purified from biopsies with protocol 1, 2 and 3 and DNA purified by the three different DNA extraction kits (kit 1–3) after enzymatic and mechanical pre-treatment (Table 1). For protocol 2 and 3 technical replicates were used. The 16S cDNA cloning and sequencing was performed on cDNA being synthesised from RNA purified from one biopsy using protocol 2. Two different cDNA synthesis reactions, containing either gene specific primer or random hexamers as described above, were used in the cloning and sequencing reactions (Table 1).

### Taxonomic assignment and data analyses

The sequences were analysed using Sequencher v/5.2 (Gene Codes Corporation, Ann Arbor, MI, USA). Chimera checking was performed using USEARCH 6.0. [19]. The sequences were taxonomically classified using Classifier, a naïve Bayesian classifier (http://rdp.cme.msu.edu/classifier/classifier.jsp) [20], and the 16S rRNA training set 14 (accession date 7 Apr 2016). Comparison analysis of DNA purified using different purification protocols and comparison analysis of cDNA synthesized using different oligonucleotides, were performed using the Library Compare Tool, with default settings, found at http://rdp.cme.msu.edu/comparison (accession date 7 Apr 2016) and described in the paper of Wang et al. [20]. Shannon diversity indexes were calculated to measure the richness and eveness for the libraries using the Shannon & Chao index tool from the RDP Pipeline (https://pyro.cme.msu.edu/index.jsp) and a 3 % distance [20] (accession date 7 Apr 2016). Rarefaction curves were calculated using the Rarefraction tool (https://pyro.cme.msu.edu/rarefaction/form.spr) with default settings (accession date 4 May 2016).

## Results and discussion

We have developed further a commercial nucleic acid protocol for efficient and simultaneous purification of microbial and human RNA and DNA from a single mucosal colonic tissue sample. The protocol is based upon widely used methods and allows for the combined study of metabolically active, latent and dead bacteria in and on the colonic mucosa. The isolated RNA and DNA will be the total RNA and DNA of the sample, thereby also offering a unique opportunity to investigate microbiota-host interactions in a single mucosal biopsy as well as micro-eukaryotes and viruses.

### Nucleic acid purification: protocol 1 and 2 and standard purification methods

Isolating RNA and DNA from a wide range of bacteria from one tissue sample can be challenging as some bacterial orders and species have ‘hard-to-lyse’ cell walls, making rough pre-treatment necessary. Other bacteria will lyse rapidly and exposure of their free nucleic acids to rough treatment can result in RNA and DNA fragmentation. We started by comparing two protocols, protocol 1 and 2 (Table 1; Fig. 1; Additional file 1) on tissue biopsies from two patients. In protocol 1, the tissues were homogenized by bead beating three times in lysis buffer RLT Plus before proceeding with RNA and DNA purification by AllPrep DNA/RNA Mini Kit (Qiagen) using kit instructions. In protocol 2 the lysates were replaced with fresh buffer RLT Plus between each round of bead beating and finally the lysates were combined before proceeding with RNA and DNA purification following the kit protocol. In the latter procedure we hypothesised that the free nucleic acids released to the lysate after each bead beating would be more protected as it was not being exposed to additional bead beating.

**Fig. 3:**
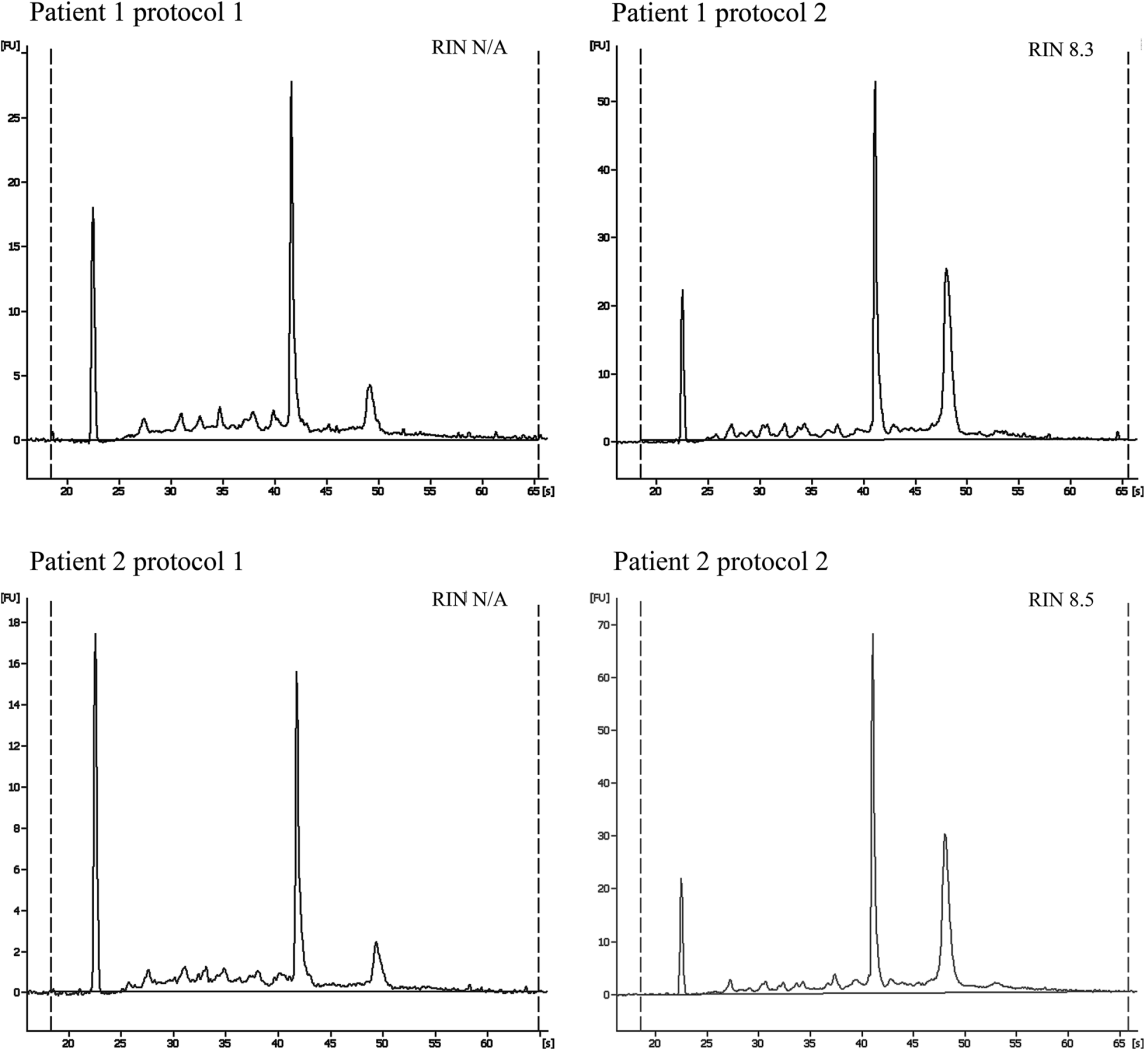
RNA quality values for purification using protocol 1 and 2. Bioanalyser electropherograms of RNA isolated using protocol 1 and 2. Fragment peaks are eukaryotic 18S and 28S

The quantity and quality of DNA was comparable between the protocols 1 and 2 as assessed by NanoDrop (Table 1). Agarose gel electrophoreses indicated that both protocols gave high molecular genomic DNA (Fig. 2). The DNA from protocol 1 showed signs of fragmentation indicating that the DNA was affected to a certain extent by the high degree of rough pre-treatment. For RNA the quantity was comparable between the protocols 1 and 2 as assessed by NanoDrop (Table 1). For the quality assessments no RIN could be calculated for neither of two RNA samples obtained from protocol 1 due to the reduction in peak height for the 28S fragment (Table 1; Fig. 3). The peak height reduction in 28S could indicate a fragmentation of the RNA when exposing the free nucleic acids to repeated bead beating. Another reason could be biological difference between two tissue biopsies collected from one person, resulting in RNA from one of the biopsies having good quality and from the second a less optimal quality. The observation of the same pattern in two individuals favours the treatment during RNA purification being responsible for the peak height reduction. The number of samples is limited and a certain conclusion cannot be drawn. The peak height reduction of RNA 28S after being exerted to protocol 1 however, indicate that the RNA is of less good quality compared to the RNA purified using protocol 2. The importance of high RIN values is discussed in literature and varies according to downstream analysis [21, 22]. A low RIN value would possibly not influence a 16S cDNA sequencing analysis, but a high quality RNA sample is preferable to keep the RNA suitable for most downstream analyses.

**Fig. 4:**
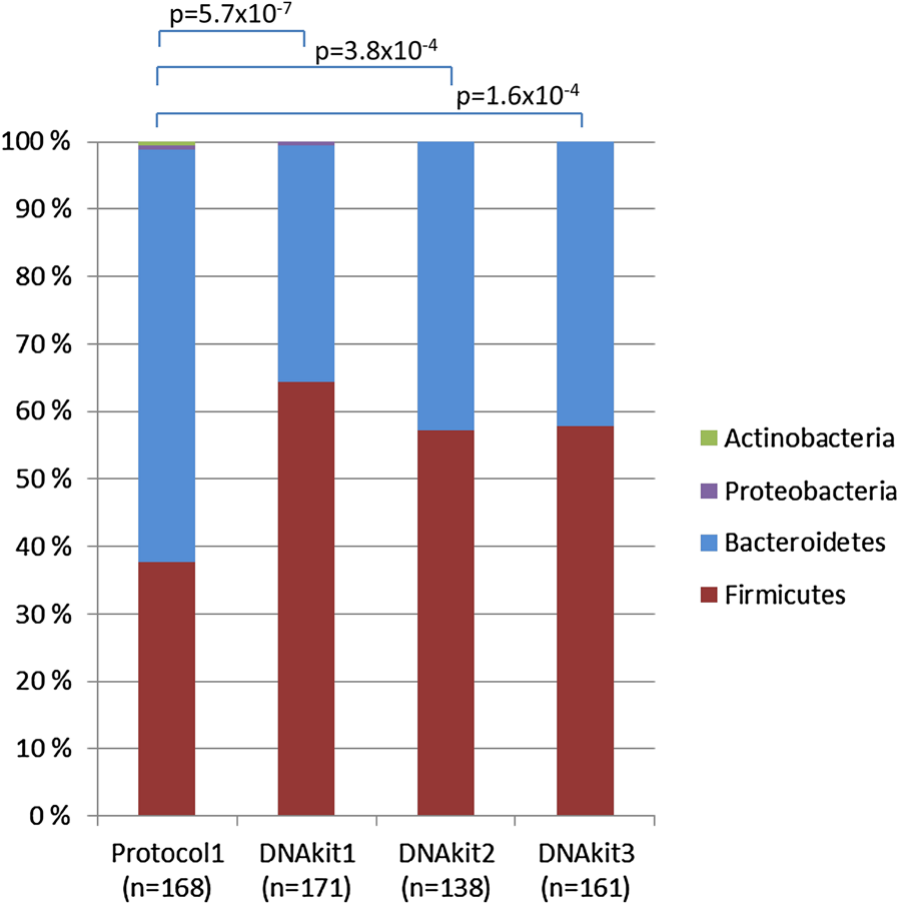
Comparison on taxonomic level of DNA purification using protocol 1 and commonly used DNA purification methods. The 16S sequence comparison bar charts are made using Classifier (http://rdp.cme.msu.edu/classifier/classifier.jsp). The significant differences of Firmicutes between paired libraries were calculated by the Library Compare Tool using a confidence threshold of 80 % (http://rdp.cme.msu.edu/comparison). Protocol 1: Mechanical pre-treatment only, followed by purification with AllPrep DNA/RNA Mini Kit as described in Additional file 1; DNA kit 1, 2 and 3: A combination of mechanical and enzymatic pre-treatments as recommended by Qiagen for lysis of Gram-positive bacteria, followed by purificaton with AllPrep DNA/RNA Mini Kit (kit 1), QIAamp DNA Stool Mini Kit (kit 2) and DNeasy Blood & Tissue Kit (kit 3) as described in Additional file 1. *n* number of clones sequenced

For purification of microbial DNA, enzymatic lysis is recommended for effective disruption of bacterial cell walls [11]. The quantity and quality of DNA purified using bead beating only (protocol 1 and 2) was comparable to DNA purified using a combination of bead beating and enzymatic lysis for all three DNA purification kits tested (kit 1–3) (Table 1). However, each method favoured different bacterial phyla. Bead beating and enzymatic lysis in combination with the AllPrep DNA/RNA Mini Kit showed to be superior in breaking the ‘hard-to-lyse’ cell walls of the Firmicutes phylum (Fig. 4).

RNA purification using a combination of Qiazol, or other chaotropic solutions, phase separation and purification kit columns, is a standard method for different types of sample material [16]. The quality of RNA using the standard RNA purification method had comparable values to RNA isolated using protocol 2, supporting protection of RNA in protocol 2 (Table 1; Additional file 2). Due to shortage of biopsies from patient 1 and 2 this comparison was performed on patient 3 using four biopsies, two biopsies per method tested (Table 1).

### Nucleic acid purification: protocol 3

The microbial and human RNA is prone to be degraded by RNases present in the human tissue. In the tissue lysis protocol of the AllPrep DNA/RNA Mini Kit, RNases and other proteins are efficiently denatured by β-mercaptoethanol added in the buffer RLT Plus. The finding of bead beating and enzymatic lysis in combination with the AllPrep DNA/RNA Mini Kit being superior in breaking the ‘hard-to-lyse’ cell walls of the Firmicutes phylum (Fig. 4) resulted in development of protocol 3 in an attempt to use enzymatic lysis and bead beating and still preserve microbial and human RNA. Protocol 3 is a combination of protocol 2 and a modified version of the enzymatic lysis procedure for stool samples published by Franzosa and colleagues [23]. The enzymatic lysis step was performed after the first two rounds of mechanical tissue lysis and buffer replacement when RNases present in the human tissue would have been denatured. The β-mercaptoethanol in buffer RLT Plus will denature lysis enzymes and the remains of the buffer RLT Plus had to be washed out of the beads, tissue debris and unlysed bacterial cells. This was performed as described in protocol 3 (Additional file 1). The enzymatic lysis step did not affect the RNA quality, suggesting that tissue RNases had been degraded in the first part of the tissue lysis protocol and that the proteinase K added together with the lysis enzymes protected the RNA from further degradation (Table 1).

**Fig. 5:**
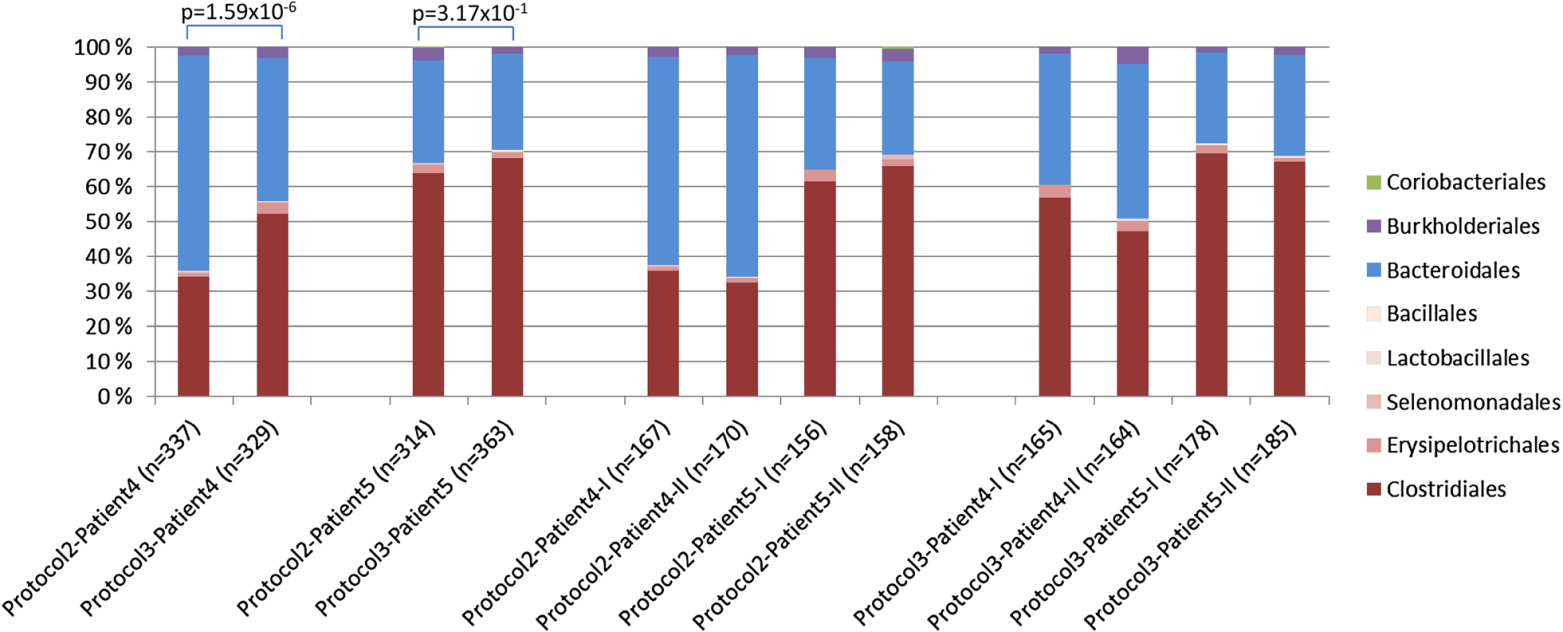
Comparison on taxonomic level of DNA purification using protocol 2 and 3. The 16S sequence comparison bar charts are made using Classifier (http://rdp.cme.msu.edu/classifier/classifier.jsp). The significant differences of Firmicutes between paired libraries were calculated by the Library Compare Tool using a confidence threshold of 80 % (http://rdp.cme.msu.edu/comparison). *n* number of clones sequenced

The comparison of microbial DNA and RNA purified from nine biopsies from four patients by cloning and sequencing resulted in a total library of 2297 sequences. A total of 59 putative chimeric sequences, identified by USEARCH, were removed from the data set (Additional file 2). To estimate the relative diversity of DNA libraries achieved by protocol 2 and 3 for each patient, we calculated the Shannon index. The microbiota diversity was not found to be significantly different between purification protocol 2 (mean 3.87 ± 0.03) and protocol 3 (mean 4.04 ± 0.18). As demonstrated by the rarefaction curves, the number of sequences analysed from each library was adequat for comparisons at order level (Additional file 2).The Library Compare tool was used to discover which bacterial groups were differentially abundant between the the libraries of protocol 2 and 3. The analyses indicated that for patient 4 a robust statistically significant difference between the the two libraries existed (p = 1.59 × 10^−6^), whereas no significant difference was found for patient 5 (p = 3.17 × 10^−1^) (Fig. 5). Interestingly, the importance of enzymatic treatment (protocol 3) seems to decrease with increasing abundance of Firmicutes. Similar results have also been observed when comparing DNA libraries from protocol 2 with DNA purified by the AllPrep standard protocol in combination with enzymatic pre-treatment (data not shown).

The DNA purified by protocol 3 showed consistency with the standard DNA purification methods using enzymatic lysis as a first step in the protocol (and hence no co-purification of RNA) followed by bead beating, regarding the relative amount of the bacterial phylum Firmicutes (Fig. 5). The benefit from protocol 3 is clear from patient 4 where the Firmicutes content is changed from 35 to 50 %. The benefit is less clear from patient 5, nevertheless no loss of Firmicutes is observed. We therefore argue that as a total, protocol 3 will insure a capture of as much of the ‘hard-to-lyse’ bacteria as possible.

### cDNA synthesis

**Fig. 6:**
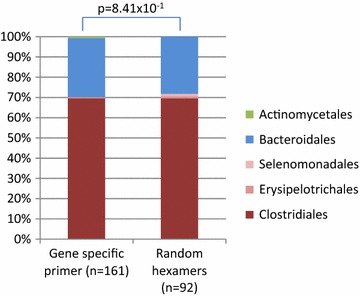
Comparison on taxonomic level of 16S cDNA. Comparison of cDNA synthesis using random hexamers and gene specific primer for RNA purification protocol 2. The 16S sequence comparison bar charts are made using Classifier (http://rdp.cme.msu.edu/classifier/classifier.jsp). The significant differences of Firmicutes between paired libraries were calculated by the Library Compare Tool using a confidence threshold of 80 % (http://rdp.cme.msu.edu/comparison). *n* number of clones sequenced

16S cDNA amplicon sequencing is performed to investigate the metabolically active members of the microbiota. In a human tissue sample most RNAs will be of human origin and the use of random hexamers in the cDNA synthesis could result in synthesis of human RNA at the expense of microbial RNA. However, the use of 16S gene specific primer will lead to a selective synthesis of cDNA and the selection depends on the primer used [24–26]. In the present study RNA purified from patients 1, 2 and 3 using protocol 2 were subjected to cDNA synthesis. Real time PCR showed lack of contaminating genomic DNA in the RNA samples. Both gene specific primer and random hexamers were used in the cDNA synthesis to investigate possible differences in the efficiency of the cDNA synthesis reaction and to investigate the bacterial taxonomic distribution obtained from the two reactions. The cDNA synthesized with gene specific primer was found to be more efficient than using random primers as illustrated in the real-time PCR results (Additional file 2). This shows that the use of gene specific primer will result in selective cDNA synthesis and that human RNA is synthesized to cDNA at the expense of microbial RNA using random primers. We found that both primer types revealed approximately the same bacterial taxonomic distribution after cloning and sequencing (Fig. 6). Due to the limited number of sequences in this analysis a recommendation is to keep in mind the selective process of a gene specific primer in the cDNA synthesis and perform a test run or pilot study before running an experiment.

Our study has additional limitations. The number of biopsies from each patient in the study is limited. This hindered the testing of all protocols on the same patient material. In addition this limitation resulted in lack of biological replicates in the study.

## Conclusions

The results of the present work give a unique possibility to study RNA and DNA from the same mucosal biopsy sample, making a direct comparison between metabolically active microbes and total microbial DNA. The protocol also offers an opportunity to investigate other members of a microbiota such as viruses, fungi and micro-eukaryotes in addition to the possibility to extract data on microbiota and host interactions from one single mucosal biopsy.

## Additional files

10.1186/s13104-016-2110-7 Purification protocols. Detailed description of the different protocol methods and the comparative DNA and RNA purification methods.
10.1186/s13104-016-2110-7 Additional results. Real-time PCR results of16S cDNA and human ß-actin cDNA (**Table S2.1, S2.2**); Hierarchial classification of cloned sequences (**Table S2.3**). **Figure S2.1.** RNA quality values for purification using protocol 2 and the standard protocol. Comparison of bioanalyser electropherograms of RNA isolated using protocol 2 and the standard RNA purification protocol. Fragment peaks are eukaryotic 18S and 28S. **Figure S2.2.** Real time PCR amplification plot of 16S cDNA synthesised using gene specific primer (926R) and random hexamers. **Figure S2.3.** Real time PCR amplification plot of human cDNA using primers targeting β-actin. **Figure S2.4.** Rarefraction analysis of clone libraries from Protocol 2 and 3 for different clustering criteria.

## Abbreviations

IBD: inflammatory bowel diseases
CD: Crohn’s disease
DNA: deoxyribonucleic acid
RNA: ribonucleic acid
RIN: RNA integrity number
cDNA: complementary DNA
NFW: nuclease free water
ACTB: β-actin
PCR: polymerase chain reaction
