# Supplementary material for: Simultaneous purification of DNA and RNA from microbiota in a single colonic mucosal biopsy
Source: BMC Res Notes. 2016 Jun 28;9:328. doi: 10.1186/s13104-016-2110-7 (PMC4924232; doi:10.1186/s13104-016-2110-7)
Supplement: Supplementary file 1 — 10.1186/s13104-016-2110-7 Purification protocols. Detailed description of the different protocol methods and the comparative DNA and RNA purification methods. [file 13104_2016_2110_MOESM1_ESM.docx]

Additional file 1

*Purification protocols*

For all protocols the biopsies were placed in 2ml sterile micro tubes (Sarstedt, Nümbrecht, Germany) containing 1pcs 5mm steal bead (Qiagen, Hilden, Germany) and 300mg 0.1mm zirconium-silica beads (Strateck, Suffolk, UK). All bead beating steps were performed using FastPrep®-24 (MP Biomedicals, Santa Ana, CA, US) with the settings 6 m/s and 30 sec.

**Protocols 1-3**

Nucleic acid purification kit: AllPrep DNA/RNA Mini Kit (Qiagen, Hilden, Germany).

1. **Extended homogenization**
2. Add 600µl Buffer RLT Plus
3. First bead beating, settings described above
4. Incubate the lysate in RT for 5 min
5. Repeat step b) and c) ) twice for a total of three rounds of bead beating
6. Centrifuge the lysate (14.000 g, 1 min, room temperature (RT))
7. Centrifuge the lysate supernatant from step e) through one DNA column
8. Follow the manufacturer’s instructions for DNA and RNA isolation
9. **Extended homogenization with replacement of buffer**
10. Add 600µl Buffer RLT Plus
11. First bead beating, settings described above
12. Centrifuge the lysate (14.000 g, 1 min, RT) and replace 400µl lysate with equal amounts of buffer RLT Plus. Leave the lysate in RT.
13. Repeat step b) and c) ) twice for a total of three rounds of bead beating
14. Centrifuge lysate supernatants from step c) through one DNA column
15. Follow the manufacturer’s instructions for DNA and RNA isolation
16. **Extended homogenization with replacement of buffer and enzymatic treatment at 25^o^C**
17. Add 600µl Buffer RLT Plus
18. First bead beating, settings described above
19. Centrifuge the lysate (14.000 g, 1 min, RT) and replace 400µl lysate with equal amounts of buffer RLT Plus. Leave the lysate in RT
20. Second bead beating, settings described above
21. Centrifuge the lysate (14.000 g, 1 min, RT). Remove 400µl lysate and leave in RT
22. Remove the remaining buffer RLT Plus in the bacterial tubes by washing with 1ml lysis buffer (30 mM Tris HCl, pH 8, 0.1 mM EDTA). Centrifuge at 14.000g, 1 min, RT and remove the supernatant. Perform wash step twice
23. Add 20µl >600 mAU/ml proteinase K (Qiagen), 20µl 100mg/ml lysozyme (Sigma-Aldrich), 15µl 1 kU/ml mutanolysin (Sigma-Aldrich) and 30µl lysis buffer to the bacterial tube. Incubate at 25^o^C, 10 min and vortex every 2 min. Add 1ml buffer RLT Plus
24. Third bead beating, settings described above
25. Centrifuge the lysate (14.000 g, 1 min, RT). Remove 1000µl lysate
26. Centrifuge lysate supernatants from step c), e) and i) through one DNA column
27. Follow the manufacturer’s instructions for DNA and RNA isolation

**DNA purification using commercial kits and tissue homogenization with enzymatic treatment**

Nucleic acid purification kit: AllPrep DNA/RNA Mini Kit (Qiagen), QIAamp DNA Stool Mini Kit (Qiagen), DNeasy Blood & Tissue Kit (Qiagen)

1. Add 252µl lysozyme buffer (25mM Tris HCl pH8, 2,5mM EDTA, 1% Triton X-100)
2. Bead beating, settings described above
3. Add 30µl 10mg/ml lysozyme (Sigma-Aldrich, St. Louis, MO, USA) 15µl 1kU/ml mutanolysin (Sigma-Aldrich) and 3µl 5 mg/ml lysostaphin (Sigma-Aldrich) and incubate for 30min at 37^o^C
4. Add excess of the respective kit lysis buffer and follow the manufacturer’s instructions for the respective kit

**Standard RNA purification method**

Trizol, phase separation and RNeasy Mini Kit (Qiagen) [1]

1. Add 1ml Qiazol (Qiagen)
2. First bead beating, settings described above
3. Centrifuge the lysate (14.000 g, 1 min, 4^o^C) and replace 600µl Qiazol-lysate with equal amounts of Qiazol. Leave the lysate on ice.
4. Repeat step b) and c) three times
5. Add 0.2 volume of chloroform to each of the three lysates and mix by vortexing. Incubate at RT for 2 minutes
6. Centrifuge (14.000 g, 15 min, 4^o^C)
7. Remove the aqueous phase and mix with 1 volume of 70 % EtOH
8. Transfer the aqueous phase from all three lysates to a single RNA Mini Spin column (Qiagen)
9. Further processing was performed as described in the RNeasy Mini Kit protocol with DNase treatment
10. Elute RNA in 40µl NFW and store at -80^o^C.

**References**

1. Wei JS, Khan J: **Purification of total RNA from mammalian cells and tissues**. In: *DNA microarrays: A molecular cloning manual.* Edited by Bowtell D, Sambrook J. Cold Spring Harbor, NY: Cold Spring Harbor Laboratory Press; 2002: 110-119.
