# Supplementary material for: Simultaneous purification of DNA and RNA from microbiota in a single colonic mucosal biopsy
Source: BMC Res Notes. 2016 Jun 28;9:328. doi: 10.1186/s13104-016-2110-7 (PMC4924232; doi:10.1186/s13104-016-2110-7)
Supplement: Supplementary file 2 — 10.1186/s13104-016-2110-7 Additional results. Real-time PCR results of16S cDNA and human ß-actin cDNA (Table S2.1, S2.2); Hierarchial classification of cloned sequences (Table S2.3). Figure S2.1. RNA quality values for purification using protocol 2 and the standard protocol. Comparison of bioanalyser electropherograms of RNA isolated using protocol 2 and the standard RNA purification protocol. Fragment peaks are eukaryotic 18S and 28S. Figure S2.2. Real time PCR amplification plot of 16S cDNA synthesised using gene specific primer (926R) and random hexamers. Figure S2.3. Real time PCR amplification plot of human cDNA using primers targeting β-actin. Figure S2.4. Rarefraction analysis of clone libraries from Protocol 2 and 3 for different clustering criteria. [file 13104_2016_2110_MOESM2_ESM.docx]

Additional file 2

Table S2.1 Real time PCR amplification results from cDNA synthesized using gene specific, random hexamers and 16S-primers [1]

| SDS 2,4 | Absolute QuantificationResults | 1 |  |  |  |
| --- | --- | --- | --- | --- | --- |
| Filename | 16S |  |  |  |  |
| PlateID |  |  |  |  |  |
| Assay Type | Absolute Quantification |  |  |  |  |
| Run DateTime | 3/30/16 4:33:45 PM |  |  |  |  |
| Operator |  |  |  |  |  |
| ThermalCycleParams |  |  |  |  |  |
|  |  |  |  |  |  |
| Sample Information |  |  |  |  |  |
|  |  |  |  |  |  |
| Position | Sample^1,2^ | Detector | Task | Ct | Ct mean |
| A1 | P3_4_RP_16_1 | 16s sybr | Unknown | 29,214 | 29,563 |
| A2 | P3_4_RP_16_1 | 16s sybr | Unknown | 31,009 |  |
| A3 | P3_4_RP_16_1 | 16s sybr | Unknown | 28,466 |  |
| A4 | P3_4_GS_16_1 | 16s sybr | Unknown | 22,319 | 22,969 |
| A5 | P3_4_GS_16_1 | 16s sybr | Unknown | 24,708 |  |
| A6 | P3_4_GS_16_1 | 16s sybr | Unknown | 21,879 |  |
| A10 | K1 | 16s sybr | Unknown | Undetermined |  |
| A11 | K1 | 16s sybr | Unknown | Undetermined |  |
| A12 | K1 | 16s sybr | Unknown | Undetermined |  |
| B1 | P3_2_RP_16_1 | 16s sybr | Unknown | 29,641 | 29,241 |
| B2 | P3_2_RP_16_1 | 16s sybr | Unknown | 29,083 |  |
| B3 | P3_2_RP_16_1 | 16s sybr | Unknown | 28,999 |  |
| B4 | P3_2_GS_16_1 | 16s sybr | Unknown | 22,613 | 22,738 |
| B5 | P3_2_GS_16_1 | 16s sybr | Unknown | 22,857 |  |
| B6 | P3_2_GS_16_1 | 16s sybr | Unknown | 22,744 |  |
| C1 | P1_RP_16_1 | 16s sybr | Unknown | 26,338 | 26,122 |
| C2 | P1_RP_16_1 | 16s sybr | Unknown | 25,614 |  |
| C3 | P1_RP_16_1 | 16s sybr | Unknown | 26,415 |  |
| C4 | P1_GS_16_1 | 16s sybr | Unknown | 20,000 | 19,918 |
| C5 | P1_GS_16_1 | 16s sybr | Unknown | 20,029 |  |
| C6 | P1_GS_16_1 | 16s sybr | Unknown | 19,726 |  |
| C10 | K2 | 16s sybr | Unknown | Undetermined |  |
| C11 | K2 | 16s sybr | Unknown | Undetermined |  |
| C12 | K2 | 16s sybr | Unknown | Undetermined |  |
| D1 | P2_RP_16_1 | 16s sybr | Unknown | Undetermined |  |
| D2 | P2_RP_16_1 | 16s sybr | Unknown | 29,966 | 29,871 |
| D3 | P2_RP_16_1 | 16s sybr | Unknown | 29,776 |  |
| D4 | P2_GS_16_1 | 16s sybr | Unknown | 23,591 | 22,994 |
| D5 | P2_GS_16_1 | 16s sybr | Unknown | 22,780 |  |
| D6 | P2_GS_16_1 | 16s sybr | Unknown | 22,610 |  |
| E1 | P3_4_RP_16_2 | 16s sybr | Unknown | 27,008 | 26,789 |
| E2 | P3_4_RP_16_2 | 16s sybr | Unknown | 26,554 |  |
| E3 | P3_4_RP_16_2 | 16s sybr | Unknown | 26,804 |  |
| E4 | P3_4_GS_16_2 | 16s sybr | Unknown | 21,499 | 21,250 |
| E5 | P3_4_GS_16_2 | 16s sybr | Unknown | 21,421 |  |
| E6 | P3_4_GS_16_2 | 16s sybr | Unknown | 20,831 |  |
| E10 | K3 | 16s sybr | Unknown | Undetermined |  |
| E11 | K3 | 16s sybr | Unknown | Undetermined |  |
| E12 | K3 | 16s sybr | Unknown | Undetermined |  |
| F1 | P3_2_RP_16_2 | 16s sybr | Unknown | 27,435 | 27,292 |
| F2 | P3_2_RP_16_2 | 16s sybr | Unknown | 27,488 |  |
| F3 | P3_2_RP_16_2 | 16s sybr | Unknown | 26,952 |  |
| F4 | P3_2_GS_16_2 | 16s sybr | Unknown | 21,120 | 21,397 |
| F5 | P3_2_GS_16_2 | 16s sybr | Unknown | 21,881 |  |
| F6 | P3_2_GS_16_2 | 16s sybr | Unknown | 21,190 |  |
| G1 | P1_RP_16_2 | 16s sybr | Unknown | 27,530 | 26,869 |
| G2 | P1_RP_16_2 | 16s sybr | Unknown | 26,508 |  |
| G3 | P1_RP_16_2 | 16s sybr | Unknown | 26,571 |  |
| G4 | P1_GS_16_2 | 16s sybr | Unknown | 20,655 | 21,638 |
| G5 | P1_GS_16_2 | 16s sybr | Unknown | 23,430 |  |
| G6 | P1_GS_16_2 | 16s sybr | Unknown | 20,829 |  |
| G10 | K4 | 16s sybr | Unknown | Undetermined |  |
| G11 | K4 | 16s sybr | Unknown | Undetermined |  |
| G12 | K4 | 16s sybr | Unknown | Undetermined |  |
| H1 | P2_RP_16_2 | 16s sybr | Unknown | 31,913 | 31,345 |
| H2 | P2_RP_16_2 | 16s sybr | Unknown | 30,935 |  |
| H3 | P2_RP_16_2 | 16s sybr | Unknown | 31,186 |  |
| H4 | P2_GS_16_2 | 16s sybr | Unknown | 24,153 | 24,341 |
| H5 | P2_GS_16_2 | 16s sybr | Unknown | 24,298 |  |
| H6 | P2_GS_16_2 | 16s sybr | Unknown | 24,573 |  |
| H10 | K5 | 16s sybr | Unknown | Undetermined |  |
| H11 | K5 | 16s sybr | Unknown | Undetermined |  |
| H12 | K5 | 16s sybr | Unknown | Undetermined |  |

1 P = Patient

RP = cDNA synthesized using random hexamers

GS = cDNA synthesized using gene specific primer

K1 = cDNA blank using gene specific primer

K2 = cDNA blank using random hexamers

K3 = minus RT control for patient 1

K4 = minus RT control for patient 2

K5 = RT-PCR negative control

2 Sample name for Patient 1 and 2 (RNA isolated from one tissue biopsy/patient):

PatientNumber_cDNAprimer(RP=RandomHexamers,GS=GeneSpecific)_16S_cDNAreplicateNumber

Sample name for patient 3 (RNA purified from two tissue biopsies)

PatientNumber_biopsyNumber_cDNAprimer(RP=RandomHexamers,GS=GeneSpecific)_16S_cDNAreplicateNumber

Table S2.2 Real time PCR amplification results from cDNA synthesized using random hexamers and ACTB-primers [2]

| SDS 2.4 | Absolute QuantificationResults | 1.0 |  |  |  |
| --- | --- | --- | --- | --- | --- |
| Filename | ACTB |  |  |  |  |
| PlateID |  |  |  |  |  |
| Assay Type | Absolute Quantification |  |  |  |  |
| Run DateTime | 04.04.2016 16:08 |  |  |  |  |
| Operator |  |  |  |  |  |
| ThermalCycleParams | |  |  |  |  |
|  |  |  |  |  |  |
| Sample Information | |  |  |  |  |
|  |  |  |  |  |  |
| Position | Sample^1,2^ | Detector | Task | Ct | Ct mean |
| A1 | P3_4_RP_ACTB_1 | SYBR | Unknown | 30,817 | 30,729 |
| A2 | P3_4_RP_ACTB_1 | SYBR | Unknown | 30,366 |  |
| A3 | P3_4_RP_ACTB_1 | SYBR | Unknown | 31,004 |  |
| A5 | K2 | SYBR | Unknown | Undetermined |  |
| A6 | K2 | SYBR | Unknown | Undetermined |  |
| A7 | K2 | SYBR | Unknown | Undetermined |  |
| B1 | P3_2_RP_ACTB_1 | SYBR | Unknown | 31,794 | 31,735 |
| B2 | P3_2_RP_ACTB_1 | SYBR | Unknown | 31,795 |  |
| B3 | P3_2_RP_ACTB_1 | SYBR | Unknown | 31,615 |  |
| C1 | P1_RP_ACTB_1 | SYBR | Unknown | 30,045 | 29,970 |
| C2 | P1_RP_ACTB_1 | SYBR | Unknown | 29,881 |  |
| C3 | P1_RP_ACTB_1 | SYBR | Unknown | 29,984 |  |
| C5 | K3 | SYBR | Unknown | Undetermined |  |
| C6 | K3 | SYBR | Unknown | Undetermined |  |
| C7 | K3 | SYBR | Unknown | Undetermined |  |
| D1 | P2_RP_ACTB_1 | SYBR | Unknown | 28,952 | 29,312 |
| D2 | P2_RP_ACTB_1 | SYBR | Unknown | 29,445 |  |
| D3 | P2_RP_ACTB_1 | SYBR | Unknown | 29,540 |  |
| E1 | P3_4_RP_ACTB_2 | SYBR | Unknown | 30,498 | 30,806 |
| E2 | P3_4_RP_ACTB_2 | SYBR | Unknown | 30,953 |  |
| E3 | P3_4_RP_ACTB_2 | SYBR | Unknown | 30,968 |  |
| E5 | K4 | SYBR | Unknown | Undetermined |  |
| E6 | K4 | SYBR | Unknown | Undetermined |  |
| E7 | K4 | SYBR | Unknown | Undetermined |  |
| F1 | P3_2_RP_ACTB_2 | SYBR | Unknown | 31,286 | 31,281 |
| F2 | P3_2_RP_ACTB_2 | SYBR | Unknown | 31,268 |  |
| F3 | P3_2_RP_ACTB_2 | SYBR | Unknown | 31,290 |  |
| G1 | P1_RP_ACTB_2 | SYBR | Unknown | 29,957 | 29,942 |
| G2 | P1_RP_ACTB_2 | SYBR | Unknown | 29,888 |  |
| G3 | P1_RP_ACTB_2 | SYBR | Unknown | 29,979 |  |
| G5 | K5 | SYBR | Unknown | Undetermined |  |
| G6 | K5 | SYBR | Unknown | Undetermined |  |
| G7 | K5 | SYBR | Unknown | Undetermined |  |
| H1 | P2_RP_ACTB_2 | SYBR | Unknown | 29,017 | 29,016 |
| H2 | P2_RP_ACTB_2 | SYBR | Unknown | 29,031 |  |
| H3 | P2_RP_ACTB_2 | SYBR | Unknown | 29,000 |  |

1 P = Patient

RP = cDNA synthesized using random hexamers

K2 = cDNA blank using random hexamers

K3 = minus RT control for patient 1

K4 = minus RT control for patient 2

K5 = RT-PCR negative control

2 Sample name for Patient 1 and 2 (RNA isolated from one tissue biopsy/patient):

PatientNumber_cDNAprimer(RP=RandomHexamers)_ACTB_cDNAreplicateNumber

Sample name for patient 3 (RNA purified from two tissue biopsies)

PatientNumber_biopsyNumber_cDNAprimer(RP=RandomHexamers)_ACTB_cDNAreplicateNumber

Table S2.3 Hierarchial classification of cloned sequences^1^

| **Taxonomical level** | **Classification** | **Number of clones** |
| --- | --- | --- |
| domain | Bacteria | 2236 |
|  | unclassified_Bacteria | 7 |
| phylum | Actinobacteria | 3 |
| class | Actinobacteria | 3 |
| subclass | Actinobacteridae | 2 |
| order | Actinomycetales | 2 |
| suborder | Frankineae | 1 |
| family | Cryptosporangiaceae | 1 |
| genus | Cryptosporangium | 1 |
| suborder | Propionibacterineae | 1 |
| family | Propionibacteriaceae | 1 |
| genus | Propionibacterium | 1 |
| subclass | Coriobacteridae | 1 |
| order | Coriobacteriales | 1 |
| suborder | Coriobacterineae | 1 |
| family | Coriobacteriaceae | 1 |
|  | unclassified_Coriobacteriaceae | 1 |
| phylum | Bacteroidetes | 888 |
|  | unclassified_"Bacteroidetes" | 1 |
| class | Bacteroidia | 887 |
| order | Bacteroidales | 887 |
|  | unclassified_"Bacteroidales" | 4 |
| family | Rikenellaceae | 21 |
| genus | Alistipes | 21 |
| family | Prevotellaceae | 69 |
| genus | Prevotella | 58 |
| genus | Paraprevotella | 11 |
| family | Bacteroidaceae | 719 |
| genus | Bacteroides | 719 |
| family | Porphyromonadaceae | 74 |
|  | unclassified_"Porphyromonadaceae" | 1 |
| genus | Butyricimonas | 2 |
| genus | Barnesiella | 26 |
| genus | Parabacteroides | 39 |
| genus | Odoribacter | 6 |
| phylum | Proteobacteria | 41 |
| class | Betaproteobacteria | 40 |
| order | Burkholderiales | 40 |
| family | Sutterellaceae | 40 |
| genus | Sutterella | 40 |
| class | Alphaproteobacteria | 1 |
|  | unclassified_Alphaproteobacteria | 1 |
| phylum | Firmicutes | 1297 |
|  | unclassified_Firmicutes | 15 |
| class | Clostridia | 1165 |
|  | unclassified_Clostridia | 2 |
| order | Clostridiales | 1163 |
|  | unclassified_Clostridiales | 16 |
| family | Clostridiaceae 1 | 11 |
|  | unclassified_Clostridiaceae 1 | 8 |
| genus | Clostridium sensu stricto | 3 |
| family | Lachnospiraceae | 779 |
|  | unclassified_Lachnospiraceae | 269 |
| genus | Ruminococcus2 | 115 |
| genus | Dorea | 52 |
| genus | Clostridium XlVa | 29 |
| genus | Clostridium XlVb | 9 |
| genus | Blautia | 197 |
| genus | Roseburia | 15 |
| genus | Coprococcus | 24 |
| genus | Anaerostipes | 16 |
| genus | Lachnospiracea_incertae_sedis | 53 |
| family | Ruminococcaceae | 356 |
|  | unclassified_Ruminococcaceae | 26 |
| genus | Faecalibacterium | 263 |
| genus | Butyricicoccus | 14 |
| genus | Gemmiger | 25 |
| genus | Ruminococcus | 3 |
| genus | Oscillibacter | 15 |
| genus | Clostridium IV | 3 |
| genus | Flavonifractor | 6 |
| genus | Sporobacter | 1 |
| family | Peptostreptococcaceae | 1 |
| genus | Clostridium XI | 1 |
| class | Bacilli | 4 |
| order | Lactobacillales | 3 |
| family | Streptococcaceae | 3 |
| genus | Streptococcus | 3 |
| order | Bacillales | 1 |
| family | Bacillales_Incertae Sedis XI | 1 |
| genus | Gemella | 1 |
| class | Negativicutes | 46 |
| order | Selenomonadales | 46 |
| family | Veillonellaceae | 44 |
|  | unclassified_Veillonellaceae | 9 |
| genus | Megamonas | 1 |
| genus | Dialister | 1 |
| genus | Veillonella | 24 |
| genus | Megasphaera | 9 |
| family | Acidaminococcaceae | 2 |
| genus | Phascolarctobacterium | 2 |
| class | Erysipelotrichia | 67 |
| order | Erysipelotrichales | 67 |
| family | Erysipelotrichaceae | 67 |
|  | unclassified_Erysipelotrichaceae | 16 |
| genus | Turicibacter | 1 |
| genus | Erysipelotrichaceae_incertae_sedis | 15 |
| genus | Catenibacterium | 10 |
| genus | Clostridium XVIII | 25 |
| no rank | Root | 2237 |
|  | unclassified_Root | 1 |

1 Classifier: Naive Bayesian rRNA Classifier Version 2.10, October 2014 .Taxonomical Hierarchy: RDP 16S rRNA training set 14. Query File: merged_alignment_total_files.fasta. Submit Date: Thu Apr 07 11:08:13 EDT 2016. Confidence threshold: 80%. CopyNumber Adjusted: no

Figure S2.1 RNA quality values for purification using protocol 2 and standard protocol

Bioanalyser electropherograms of RNA isolated using protocol 2 and standard protocol. Fragment peaks are eukaryotic 18S and 28S


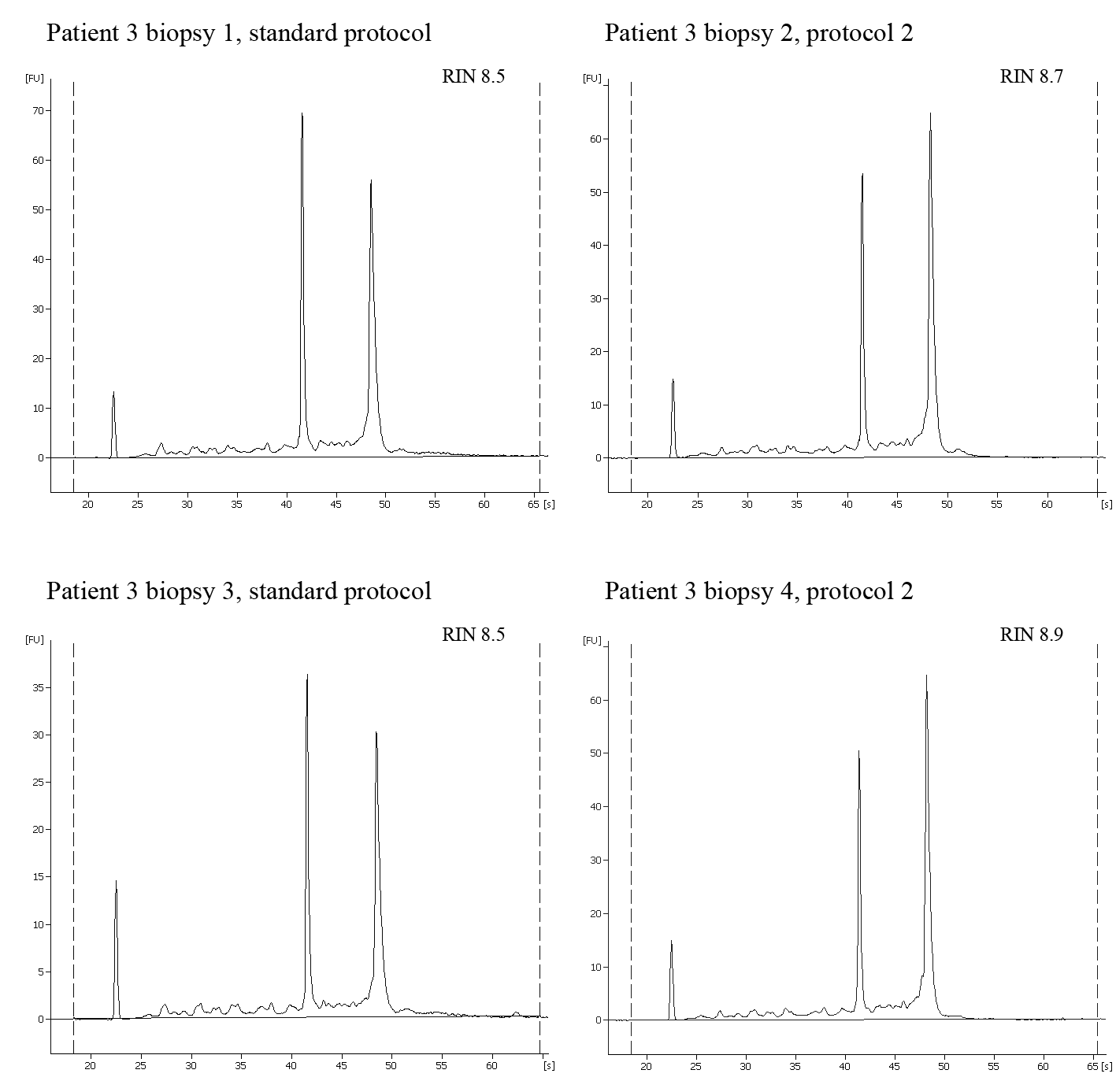


Figure S2.2 Real time PCR amplification plot, 16S cDNA synthesised using gene specific primer (926R) and random hexamers

**
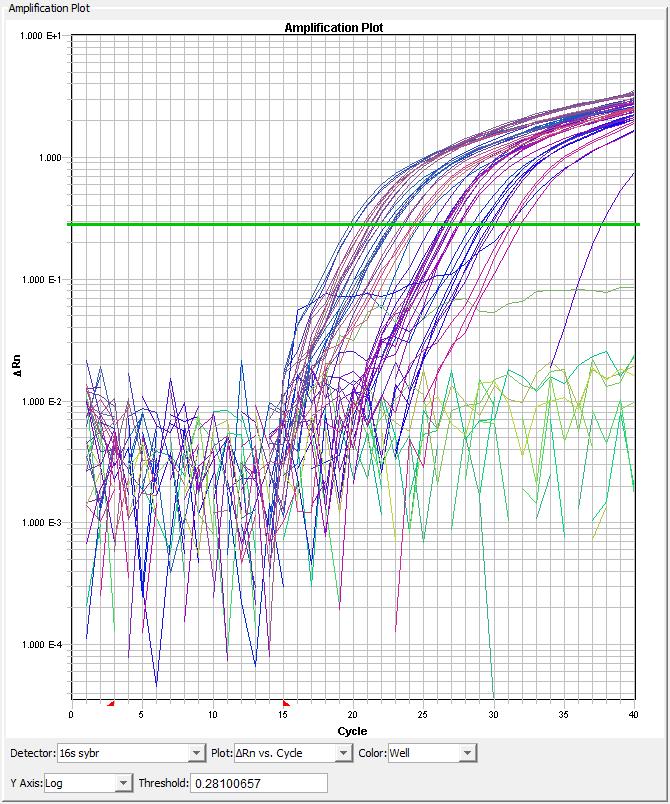
**

Figure S2.3 Real time PCR amplification plot, human cDNA using primers targeting β-actin

**
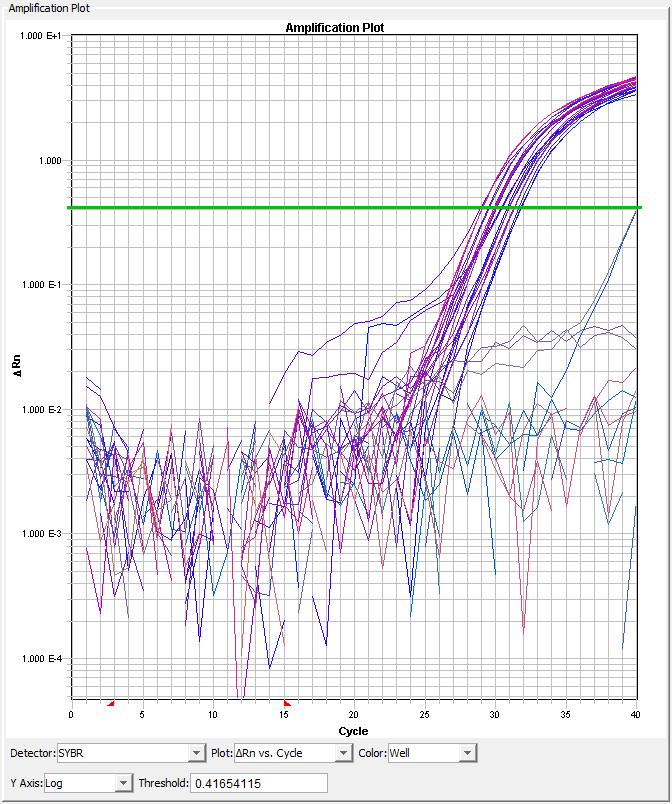
**

Figure S2.4 Rarefraction analysis of clone libraries from Protocol 2 and 3 for different clustering criteria.

# References

1. Segata N, Izard J, Waldron L, Gevers D, Miropolsky L, Garrett W, Huttenhower C: **Metagenomic biomarker discovery and explanation**. *Genome Biol* 2011, **12**(6):R60.

2. Sørby LA, Jonsdottir K, Beiske K, Blom P, Bukholm IRK, Jacobsen MB: **Cyclin A2 Protein Overexpression Is Not Caused by Gene Amplification in Colon Cancer**. *ISRN Pathology* 2012, **2012**:7.
